# Mountain grasslands as carbon sinks and energy hubs: a study of Western Carpathians in Slovakia

**DOI:** 10.1038/s41598-025-30358-x

**Published:** 2025-12-02

**Authors:** Miriam Kizeková, Norbert Britaňák, Jozef Čunderlík, Ľubomír Hanzes, Štefan Pollák, Vladimíra Vargová, Ľubica Jančová, Radoslava Kanianska

**Affiliations:** 1 National Agricultural and Food Centre, Research Institute of Plant Production, Grassland and Mountain Agriculture Institute, Mládežnícka 36, 974 21 Banská Bystrica, Slovakia; 2 Faculty of Natural Sciences, Matej Bel University Banská Bystrica, Tajovského 40, Banská Bystrica, 974 01 Slovakia

**Keywords:** Grasslands, Carbon stock, Bioenergy potential, Soil properties, Western carpathians, Environmental sciences, Climate change

## Abstract

Mountain grasslands are unique ecosystems that provide many ecosystems’ services. They are an important carbon pool and can also act as a source of bioenergy. The study focused on 4 grassland types (Lowland hay meadows, Mountain hay meadows, Wet grassland of sub-montane zones and Artificial grasslands) and 4 soil types (Fluvisols, Cambisols, Leptosols, Stagnosols) in 5 mountain ranges (Great Fatra Mts., Low Tatra Mts., Slovak Paradise Mts., Čergov Mts., Slánske Hills Mts.) of the Western Carpathians in Slovakia. The results showed that of the total carbon stock of the mountain ecosystems equal to 2.120 × 10^3^ tons, 94% was stored in soil. The total carbon density followed this pattern: Fluvisols (100.64 ± 7.87 t/ha) > Stagnosols (84.17 ± 7.90 t/ha) > Leptosols (56.34 ± 6.20 t/ha) > Cambisols (55.43 ± 5.09 t/ha). Soil organic carbon density was also significantly affected by elevation, with the highest values between 600 and 900 m asl. (70.46 ± 4.23 t/ha). Grassland type significantly affected carbon density in living plant biomass, where Mountain hay meadows showed the significantly lowest values (*P* < 0.05) for carbon density in above-ground biomass (0.79 ± 0.05 t/ha) and below-ground biomass (2.24 ± 0.16 t/ha). The hierarchical clustering dendrogram revealed that the soils from the Low Tatra Mts. differed significantly from those of other mountain ranges and were characterized by high pH, very high soil organic carbon content, high levels of plant-available magnesium, and high C: N ratio in the soil. The calculated biomethane production ranged from 6,606 GJ in the Slovak Paradise Mts. for Lowland hay meadows, to 122,888 GJ in the Low Tatra Mts., which had the largest area of grassland habitats. The total theoretical energetic potential of biomethane production from all evaluated grassland habitats exceeded 1,139 GJ. The energetic potential values followed the following order: Slovak Paradise Mts. < Čergov Mts. < Slánske Hills Mts. < Great Fatra Mts. < Low Tatra Mts.

## Introduction

 Grasslands cover up to 40% of the terrestrial area and they are found worldwide in a variety of climatic regimes, except for regions characterized by extreme aridity and the highest mountains^1^. They provide numerous ecosystem services e.g. provisioning services such as forage for herbivores, cultural service such as recreational value, and supporting service such as biodiversity^2^. Among these, regulating services—especially carbon storage, erosion control, and water regulation—have gained increasing attention due to their contribution to climate mitigation and landscape stability^3–5^.

Carbon cycling in grasslands involves both above-ground and below-ground components. Above-ground biomass plays a vital role in the global carbon cycle^6^ while variation in biomass carbon density is driven by environmental factors such as soil properties, topography, and climatic conditions^7–9^. Because a major portion of photosynthetically fixed carbon is allocated below ground, roots play a key role in carbon sequestration^10,11^.

Many studies have been conducted on total carbon stock in various ecosystems including forests^12,13^, grasslands^14^, shrublands^15^, and agricultural land^16^. Research on grasslands has primarily focused on evaluating how grassland type^17^, altitudinal gradients^18^, and management practices^19,20^ such as grazing intensity, cutting frequency, fertilization, liming, burning, irrigation, and the use of grass–clover mixtures, affect total carbon stocks. However, relatively few studies have examined total carbon stocks specifically in mountain grasslands^21,22^, despite their ecological importance and vulnerability.

Slovakia is a predominantly mountainous country where permanent grasslands cover 17% of the territory^23^, and extensive areas of species-rich mountain meadows and pastures occur within forested landscapes. These habitats provide both biodiversity and climate-regulating functions and offer potential feedstock for bioenergy production^24^. The European Union’s long-term climate neutrality goals and the Renewable Energy Directive highlight the need to support renewable energy deployment while safeguarding sensitive habitats^25^. Understanding the carbon and energy potential of mountain grasslands is therefore important for sustainable management. While carbon stock assessments exist for Slovak agricultural soils^26^ and some forest ecosystems^27^, comprehensive analyses of total carbon stock and biomethane potential of mountain grasslands are still lacking.

The general aim of this study is to improve knowledge on carbon stocks and the energy potential of Slovak mountain grasslands, thereby contributing to sustainable management and climate change adaptation. In this context, energy potential refers to the theoretical amount of energy that could be obtained from grassland biomass under optimal conditions, while *bioenergy production* (in this case, biomethane production) denotes the conversion of this biomass into usable energy forms. Specifically, the study focuses on: (i) evaluating the chemical properties of mountain soils, (ii) estimating carbon stocks across different grassland and soil types, and (iii) assessing the theoretical energy content and biomethane production potential of grasslands in five mountain ranges of the Western Carpathians. In addition, the results highlight areas with potential for biomass production and energy generation. Greater knowledge of the potential of mountain grasslands can help to address a more sustainable and climate-resilient future.

## Materials and methods

### Description of study sites

**Fig. 1:**
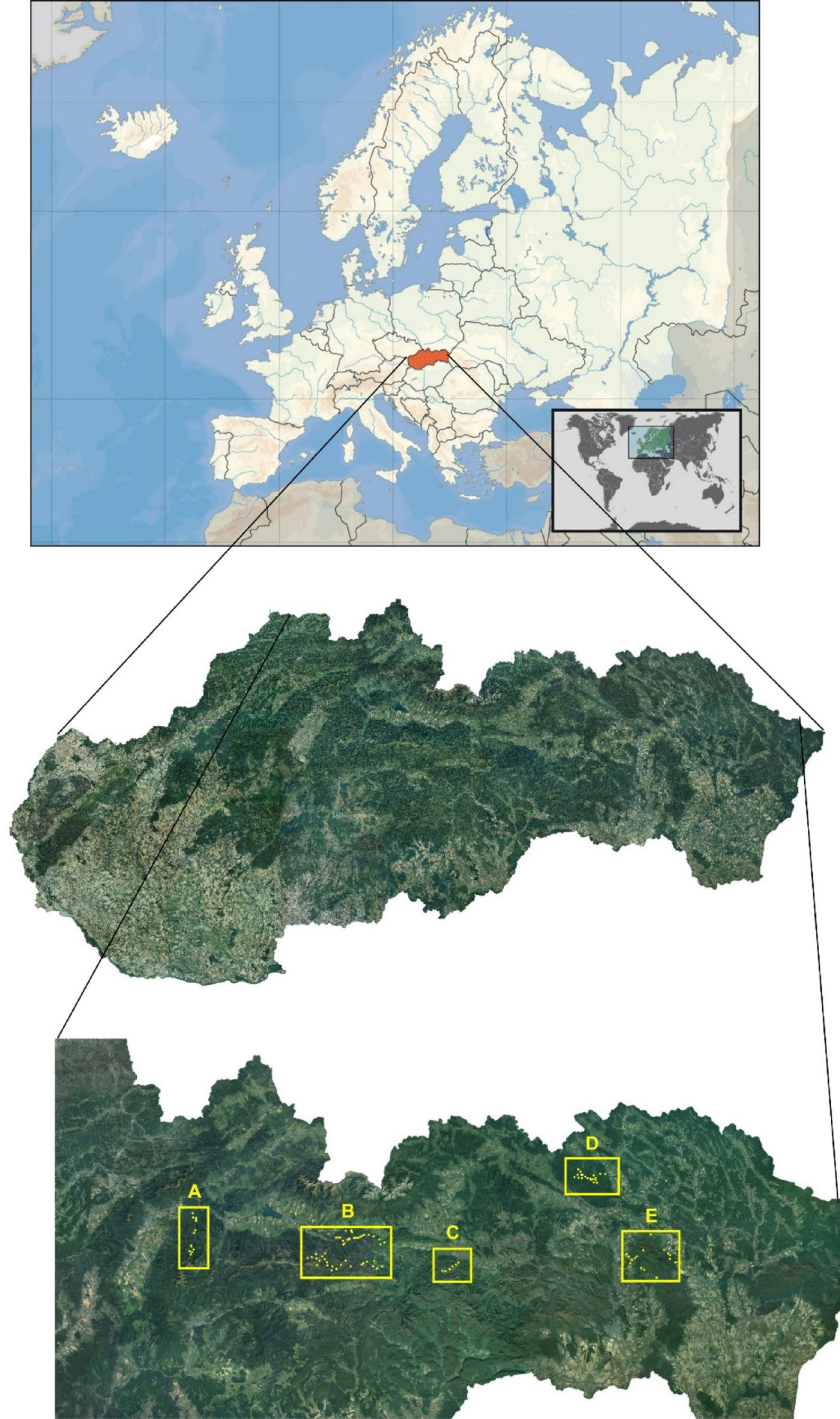
The map of Slovakia and location of sample sites in the mountain ranges of Western Carpathians : **A**) Great Fatra Mts., **B**) Low Tatra Mts., **C**) Slovak Paradise Mts., **D**) Čergov Mts., **E**) Slánké Hills Mts.

Slovakia is an inland country in Central Europe. Of the total area of 49,033 km^2^, the Western and Eastern Carpathian Mountain chain occupy more than 60%. The mountain areas are forest/grassland-dominated ecosystems with great geodiversity and biodiversity^28^. The Western Carpathians are the highest part of the Carpathians with scenic ridges separated by fluvial valleys^29^. The study was conducted in 5 mountain ranges of the Western Carpathains (Fig. 1) for 2 years in 2020 and 2021. The study consists of 4 grassland types and 4 soil types as well.

The **Great Fatra Mts**. with the area of 784 km^2^ are a part of the Inner Western Carpathians and are located west of the Low Tatras Mts. In the **Great Fatra** Mts., plots with Lowland hay meadows, Mountain hay meadows and Artificial grasslands were sampled in the cadastral area of Ľubochňa at an altitude between 509 m asl. and 834 m asl. At the sampling sites in the Greater Fatra Mountains, Rendzic Leptosols, Calcaric Fluvisols and Dystric Cambisols with clayey texture, were predominant. The skeleton content varied from 20% to 50% with Rendzic Leptosols being moderately stony^30^.

The **Low Tatras Mts**. (area of 1,258 km^2^) are located in the central part of the Western Carpathians. In Low Tatras Mts, grassland survey plots were sampled in three cadastral areas: Východná, Nižná Boca, Malužiná and Liptovská Teplička. The elevation of the survey plots ranged from 712 m asl to 1 100 m asl in the Východná cadastral area, from 727 m asl to 1 275 m asl in the Nižná Boca cadastral area and from 765 m asl to 1 275 m asl in Liptovská Teplička. In our study the following soil types were sampled in the Low Tatra Mts.: Cambisols (Stagni-Eutric Cambisols, Dystric Cambisols and Cambic Umbrisols), Fluvisols (Eutric Fluvisols and Gleyic Eutric Fluvisols), Rendzic Leptosols and Stagnosols were the dominant soils. The soil texture varied from sandy-loamy to loamy with a large variability in skeleton content from non-skeletal to very stony^30^. Lowland hay meadows, Mountain hay meadows, Wet grasslands of sub-mountain zones, and Artificial grasslands were surveyed in the Low Tatra Mts.

The area (19,736 km^2^) of the **Slovak Paradise** Mts. belongs to the Inner Western Carpathians. In cadastral area of Hrabušice at an altitude between 580 m asl and 985 m asl. Lowland hay meadows of the soil types Rendzic Leptosols, Dystric Cambisols, Calcaric Fluvisols were sampled. The soil texture was sandy-loamy or loamy with a very low or moderate gravel content^30^.

The **Čergov** Mts. are part of the Eastern section of the Western Beskids within the Western Outer Carpathians. In the cadastral area of Livovská Huta, plots with Lowland hay meadows and Mountain hay meadows were sampled at the altitude between 673 m asl and 1 015 m asl. In this mountain range, Dystric Cambisols and Stagnosols, with loamy-sandy to clay-loamy texture were sampled^30^.

The volcanic mountain range **Slanské Hills** Mts. belongs to the Mátra-Slanec Area within the Inner Western Carpathians^31^. The altitude of the surveyed plots ranged from the 420 m asl to 968 m asl. In the sampled plots in the Slanské Hills Mts., soils were classified as Stagni-Eutric Cambisols, Dystric Cambisols and^30^. In the Slánské Hills, Lowland hay meadows, Mountain hay meadows, Wet grasslands of sub-mountain zones, and Artificial grasslands were surveyed.

### Description of grassland types

Lowland hay meadows (6510 in the EU Habitats Directive Annex I) are the most abundant grassland habitat in Slovakia. It occurs on various type of soils and its elevation ranges from 200 m asl in lowlands to 1 050 m asl in mountainous areas^32^. Due to very broad environmental conditions and management, the species structure is also highly variable. In our study, the dominant species were *Festuca pratensis*, Festuca rubra, Arrhenatherum elatius.

Mountain hay meadows (6520 in the EU Habitats Directive Annex I) are species-rich mesophilous montane meadows in upland, mountain and sub-alpine mountain areas^33^. In our study, the dominant species were *Agrostis capillaris*, *Deschampsia cespitosa*, *Hypericum maculatum.*

Wet grasslands of sub-montane zones (habitat of national importance) are mostly found in stream alluvia. Their existence is conditioned by sufficient soil moisture and fluctuation of the water Table^33^. In this study, *Alopecurus pratensis*, *Cirsium rivulare*, *Filipendula ulmaria* are the dominant species.

Artificial grasslands were created in forest nurseries and in areas used for timber harvesting. The mixtures were composed of *Festuca pratensis*, *Festuca rubra*, *Trifolium pratense* and *Trifolium repens*.

### Soil chemical properties and above-ground biomass analysis

Soil and above-ground samples were collected from the same plots. In total, we collected 98 composite soil samples. Each composite sample consisted of 4 subsamples taken in a Z-shaped transect within a 10 × 10 m area. Soil samples were taken at a depth of 0–15 cm, air-dried and sieved through a 2 mm mesh sieve. Soil organic carbon content (SOC) was determined by Tjurin method and soil reaction was determined as pH in 1 M KCl solution. Total nitrogen content (NT) was determined by the Kjeldahl method^34^. Plant-available phosphorus (P), plant-available potassium (K) and plant-available magnesium (Mg) were extracted by Melich III^35^. K was determined by flame photometry, P calorimetrically on Scalar analyser, and Mg by Atomic Absorption Spectroscopy. Above-ground biomass (AGB) was clipped from 0.5 m x 0.5 m square surfaces in spring. The dry matter yield of above-ground biomass (DMY_AGB_) was determined by drying at 60 °C for 24 h.

## Data analysis of carbon density and carbon stock

### Soil carbon density

To assess the soil type, soil texture and content of gravel and stones, a publicly available digital map, the Landscape Atlas of the Slovak Republic was used (https://app.sazp.sk/atlassr/) http://www.podnemapy.sk/portal/verejnost/obj_hmotnost/obj_hmotnost.aspx.

The soil organic carbon density (C-SOC) (t/ha) in the depth 0–15 cm was calculated as follows:

$${\rm C-SOC = SOC\times\;BD \times H \times [1-(G/100)]\times10000}$$

where: SOC is soil organic carbon content (g/kg); BD is bulk density (g/cm^3^); H is the soil thickness (m); G is the volume percent of gravel.

To assess the bulk density (BD) (g/cm^3^) we used the pedo-transfer function based on the soil texture triangle and soil organic carbon content as follows:

$${\rm BD = BDm_{x} [1-(SOC/1000)] +(SOC/1000)_{x} 0.224}$$

Where: BDm is the soil mineral bulk density (g/cm^3^) according to the soil mineral bulk density triangle (http://www.podnemapy.sk/portal/verejnost/obj_hmotnost/obj_hmotnost.aspx); SOC is the soil organic carbon content (g/kg); 0.224 is the volume weight of soil organic matter (g/cm^3^).

In total 196 composite samples of above-ground biomass were taken during the period 2020 and 2021. The living plant biomass carbon density (C-LPB) was calculated as the sum of above-ground biomass carbon density (C-AGB) and below-ground biomass carbon density (C-BGB) as follows:

$${\rm C-LPB =C-AGB +C-BGB.}$$

where: C-LPB is living plant biomass carbon density (t/ha); C-AGB is above-ground biomass carbon density (t/ha); C-BGB is below-ground biomass carbon density (t/ha).

To calculate the above-ground biomass carbon density (C-ABGB) and the below-ground biomass carbon density (C-BGB) the following equations were used:

$${\rm C-AGB=DMY_{AGB}x\: 0.47}$$

where: C-AGB is above-ground biomass carbon density (t/ha); DMY_AGB_ is dry matter yield of above-ground biomass (t/ha); 0.47 is the default value of carbon per tonne of biomass^36^.

$${\rm C-BGB =BGB\: x\: 0.47}$$

where: C-BGB is below-ground biomass carbon density (t/ha); BGB is below-ground biomass (t/ha); 0.47 is the default value of carbon per tonne of biomass^36^.

Since root biomass was not sampled, the below-ground biomass was calculated using the following formula:

$${\rm BGB=DMY_{AGB}\: x\: BGB/AGB\: ratio}$$

where: BGB is below-ground biomass (t/ha); DMY_AGB_ is dry matter yield of above-ground biomass (t/ha); BGB/AGB ratio is the ratio of belowground biomass to aboveground biomass.

For Lowland hay meadows, Mountain hay meadows and Artificial grasslands, a BGB/AGB ratio of 2.8 was applied, whereas for Wet grasslands of sub-mountain zones, a BGB/AGB ratio of 4.0 was applied.

Litter carbon density (C-Litter) (t/ha) was estimated using the following equation:

$${\rm C-Litter=(DMY_{AGB}\: x \:0.3) \:x \:0.47}$$

where: C-Litter is litter carbon density (t/ha); DMY_AGB_ is the dry matter yield of above-ground biomass (t/ha); 0.3 is the default value for the litter as the above-ground residues; 0.47 is the default value for carbon per tonne of biomass^36^.

Total carbon density (C-TOT) (t/ha) was calculated as the sum of soil organic carbon density (C-SOC) (t/ha), living plant biomass carbon density (C-PLB) (t/ha) and litter carbon density (C-Litter) (t/ha) as follows:

$${\rm C-TOT=C-SOC+C-PLB+C-Litter}$$

### Data analysis of biomethane potential

Methane energy yield (MEY) was calculated as follows:

$${\rm MEY=DMY_{AGB}\: x\: SMY \:x \:37 / 1000}$$

where: MEY is methane energy yield (GJ/ha); DMY_AGB_ is dry matter yield of above-ground biomass (t/ha); SMY is substrate-specific methane yield (l_N_/kg ODS); 37 is gross calorific value of methane (MJ/m^3^) at 293.15 K and 1 atm^37^.

Gross energy yield GEY was calculated by the combination of dry matter yield of above-ground biomass (DMY_AGB_) and gross calorific value (GCV) of each grassland type as follows:

$${\rm GEY=DMY_{AGB}\: x\: GCV}$$

where: DMY_AG_ is dry matter yield of above-ground biomass (t/ha); GCV is gross calorific value (MJ/kg dry solids).

To calculate area specific methane yield (ASMY), the following equation was used:

$${\rm ASMY= DMY_{AGB}\: x\: SMY}$$

where: ASMY is area specific methane yield (m^3^ CH_4_/ha); DMY_AGB_ is dry matter yield of above-ground biomass (t/ha); SMY is substrate-specific methane yield (l_N_/kg ODS).

Since the SMY of grassland types was not determined by direct measurements, the following values were used: 122 l_N_/kg ODS, 124 l_N_/kg ODS and 143 l_N_/kg ODS Lowland hay meadows, Wet grasslands of sub-montane zones, Artificial grasslands^38^ and 120 l_N_/kg ODS for Mountain hay meadows^39^. Similarly, for GCV the following data were applied: 17.04 MJ/ha, 17.01 MJ/ha, 17.57 MJ/ha for Lowland hay meadows, Wet grasslands of sub-montane zones, Artificial grasslands^38^ and 16.80 MJ/ha for Mountain hay meadows^39^.

### Statistical analysis

Soil properties, carbon stock and biomethane potential data were evaluated using a two-way ANOVA. Post-hoc comparisons of soil types, grassland types, mountain ranges and elevation were performed using the Tuckey HSD test with the *p* < 0.05 level. Relationships between soil chemical properties and Pearson correlation coefficient were also used. Hierarchical cluster analysis (HCA) was performed to group similar soil types based on their characteristics.

## Results

### Soil nutrient content and soil reaction

In general, the plant-available P content was consistently low across the study area and did not differ among soil types, grassland types, or mountain ranges. In contrast, soil NT content showed differences among soil types (F = 4.71, *p* = 0.004), with the highest values in Leptosols. The mean plant-available Mg content was very high, but unlike to the plant-available P, it varied significantly across soil types (F = 11.65, *p* < 0.001), grassland types (F = 6.48, *p* < 0.001), and mountain ranges (F = 7.36, *p* < 0.001).

The lowest quality of soil organic matter, expressed by the soil C: N ratio, was observed in the Great Fatra Mts. (9.33 ± 1.52). Across the 98 collected plots, SOC content in the 0–15 cm soil layer showed large spatial variability. The SOC content was not significantly affected by grassland habitat (Table 1), Cambisols showed the significantly lowest SOC content. The Slovak Paradise Mts. exhibited the highest mean SOC content (59.22 ± 6.99 g/kg), whereas mountains located in eastern Slovakia (Slánske Hills Mts. and Čergov Mts.) showed values about 40% lower, underscoring the strong influence of regional geology and climate Table 1.

**Table 1:** Soil reaction (pH), content of soil organic carbon (SOC), content of total nitrogen (NT), plant available phosphorus (P), plant available potassium (K), plant available magnesium (Mg) in different soil types, grassland types and elevation. Contrasting letters denotes significant differences. P-values highlighted in bold are highly statistically significant (*P* < 0.001) and statistically significant (*P* < 0.05).

Soil type	pH	SOC	NT	*P*	K	Mg	C: *N*
Fluvisols	5.17 ± 1.04 ^ab^	48.65 ± 2.44 ^a^	3.83 ± 0.33 ^ab^	6.72 ± 3.45^a^	125.75 ± 17.82 ^ab^	424.16 ± 128.91 ^b^	13.62 ± 1.83 ^a^
Cambisols	3.90 ± 0.71 ^c^	36.33 ± 4.71 ^b^	3.16 ± 0.13 ^b^	4.67 ± 1.15^a^	168.60 ± 21.83 ^a^	219.05 ± 56.79 ^c^	11.77 ± 0.66 ^a^
Leptosols	5.56 ± 0.75 ^a^	59.22 ± 6.99 ^a^	4.67 ± 0.59 ^a^	4.14 ± 1.74^a^	109.11 ± 14.04 ^b^	723.41 ± 254.21 ^a^	11.27 ± 1.75 ^a^
Stagnosols	4.35 ± 1.21^bc^	35.99 ± 3.00 ^ab^	3.60 ± 0.57 ^ab^	5.15 ± 4.89^a^	137.00 ± 48.17 ^ab^	371.88 ± 183.73 ^ab^	12.14 ± 1.71 ^a^
F value	21.03	7.51	4.71	0.88	3.96	11.65	2.27
P value	< 0.001	< 0.001	0.004	0.454	0.010	< 0.001	0.085
Grassland type							
Lowland hay meadows	4.78 ± 1.01^a^	44.22 ± 2.74 ^a^	3.66 ± 0.22 ^a^	5.86 ± 2.19 ^ab^	145.97 ± 19.79 ^ab^	367.15 ± 96.30 ^ab^	12.21 ± 1.10 ^a^
Mountain hay meadows	3.69 ± 0.81^b^	39.64 ± 2.81 ^a^	3.64 ± 0.26 ^a^	3.56 ± 0.57 ^b^	175.64 ± 26.57 ^a^	186.04 ± 87.34 ^b^	11.38 ± 0.83 ^a^
Wet grasslands of sub-montane zones	5.11 ± 1.00	46.60 ± 4.87 ^a^	3.81 ± 0.45 ^a^	3.27 ± 0.99 ^ab^	100.51 ± 12.79 ^b^	558.83 ± 210.79 ^a^	12.58 ± 1.30 ^a^
Artificial grasslands	4.96 ± 0.81^a^	38.05 ± 4.68 ^a^	2.77 ± 0.33 ^a^	8.96 ± 4.67 ^a^	125.50 ± 40.76 ^ab^	514.95 ± 195.14 ^a^	13.87 ± 2.52 ^a^
F value	12.96	0.938	1.53	3.81	4.35	6.48	1.98
P value	< 0.001	0.4261	0.211	0.012	0.010	< 0.001	0.122
Mountain range							
Great Fatra Mts.	5.12 ± 0.87 ^a^	36.33 ± 4.71 ^ab^	4.13 ± 0.65 ^a^	6.49 ± 6.17 ^a^	123.61 ± 34.58 ^a^	411.66 ± 142.40 ^a^	9.33 ± 1.52 ^c^
Low Tatras Mts.	5.07 ± 1.06 ^ab^	48.06 ± 2.44 ^a^	3.66 ± 0.26 ^a^	5.33 ± 1.81 ^a^	106.69 ± 8.04 ^a^	489.79 ± 129.56 ^a^	13.85 ± 1.18 ^a^
Slovak Paradise Mts.	4.93 ± 0.84 ^ab^	59.22 ± 6.99 ^a^	4.10 ± 0.73 ^a^	9.56 ± 5.05 ^a^	161.74 ± 33.35 ^a^	458.31 ± 360.37 ^ab^	14.72 ± 2.59 ^ab^
Čergov Mts.	3.34 ± 0.19 ^c^	35.99 ± 3.00 ^b^	3.33 ± 0.18 ^a^	3.31 ± 0.50 ^a^	187.17 ± 29.57 ^a^	97.57 ± 19.08 ^b^	10.94 ± 0.62 ^bc^
Slánské Hills Mts.	4.28 ± 0.53^b^	36.05 ± 3.79 ^b^	3.13 ± 0.26 ^a^	5.39 ± 2.51 ^a^	191.49 ± 42.23 ^a^	341.00 ± 94.19 ^ab^	11.52 ± 1.06 ^bc^
F value	21.61	4.80	1.21	1.67	9.69	7.36	8.47
P value	< 0.001	< 0.001	0.310	0.165	0.133	< 0.001	< 0.001
Elevation							
To 600 m asl	4.68 ± 0.29 ^a^	32.22 ± 6.12 ^a^	2.99 ± 0.48 ^a^	5.21 ± 2.18 ^a^	154.02 ± 38.51^a^	407.88 ± 91.58 ^a^	10.87 ± 1.35 ^a^
From 600 m asl to 900 m asl	4.48 ± 0.29 ^a^	43.76 ± 4.20 ^b^	3.65 ± 0.37 ^a^	5.04 ± 1.45 ^a^	147.49 ± 16.92 ^a^	348.23 ± 88.39 ^a^	12.55 ± 0.83 ^a^
Over 900 m asl	4.09 ± 0.57 ^a^	47.06 ± 8.50 ^b^	3.90 ± 0.75 ^a^	5.43 ± 3.05 ^a^	143.99 ± 32.22 ^a^	251.19 ± 174.88 ^a^	12.38 ± 1.42 ^a^
F value	1.32	4.62	2.16	0.02	0.09	0.96	2.08
P value	0.269	0.012	0.120	0.971	0.910	0.382	0.130
Interactions							
Soil Type x Grassland type							
F value	2.41	2.25	1.75	0.59	0.23	0.33	1.48
P value	0.017	0.025	0.089	0.795	0.987	0.961	0.166
Soil Type x Mountain range							
F value	9.07	2.82	2.22	1.42	4.05	5.09	1.58
P value	< 0.001	0.008	0.033	< 0.001	0.198	< 0.001	0.142
Soil Type x Elevation							
F value	3.31	1.34	1.27	1.52	1.03	3.82	1.64
P value	0.008	0.253	0.283	0.190	0.405	0.003	0.155

The interaction between soil type and mountain range on SOC content was also significant (F = 2.82, *p* = 0.008). Overall, soil organic carbon content increased with elevation; however, a significantly lower SOC content was recorded at 600 m asl (32.22 ± 3.62 g/kg) compared to the higher altitudes. Above 600 m asl, no significant differences in SOC content were observed.

On average, soils were highly acidic what is consistent with leaching dominated mountain environment. The lowest soil reaction, classified as extremely acid (pH = 3.08) occured in Cambisols of the Čergov Mts. Among the grassland types, soils in Mountain hay meadows were significantly more acidic (F = 12.96, *p* < 0.001) compared with soils in Lowland hay meadows, Wet grasslands in sub-montane zones, and Artificial grasslands. Soil reaction was shaped by several interacting factors, including soil type in combination with grassland type (F = 2.41, *p* = 0.017), mountain range (F = 9.07, *p* < 0.001) and elevation (F = 3.31, *p* = 0.008).

**Fig. 2:**
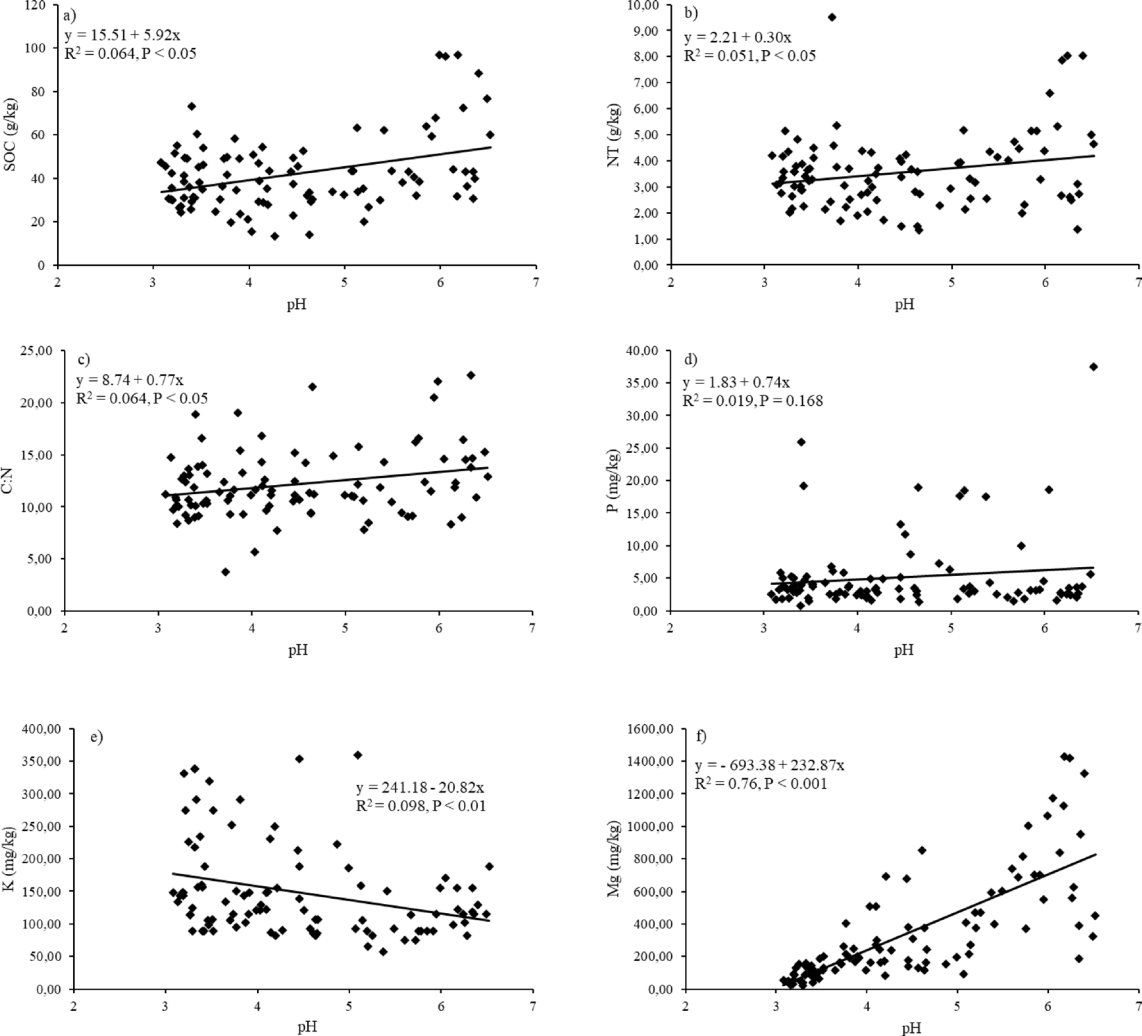
The relationship between **a**) soil pH and content of soil organic carbon (SOC), **b**) soil pH and content of total nitrogen (NT), **c**) soil pH and soil C: N, **d**) soil pH and content of plant-avaiable phosphorus (P), **e**) soil pH and content of plant-avaiable potassium (K), **f**) soil pH and plant-avaiable magnesium (Mg). The coefficient of determination (R^2^) and P value are shown in each plot.

Soil pH showed weak but a statistically significant positive correlation with SOC (R² = 0.064, *p* < 0.05) and with the soil C: N ratio (R^2^ = 0.064, *p* < 0.05). The relationship with plant-available Mg was much stronger (R^2^ = 0.571, *p* < 0.001) (Fig. 2). In contrast to Mg, plant-available K exhibited a statistically significant negative correlation with soil reaction (R^2^ = 0.098, *p* < 0.01). The positive correlation between pH and plant-available P was not statistically significant (R^2^ = 0.019, *p* = 0.168).

### Soil organic carbon density

The results showed that Fluvisols had significantly highest C-SOC (95.52 ± 7.95 t/ha), followed by Stagnosols (84.17 ± 7.90 t/ha), Leptosols (52.20 ± 6.37 t/ha), and Cambisols (55.43 ± 2.54 t/ha) (Table 2). In the case of Leptosols, C-SOC contrasted with SOC content, as C-SOC content showed statistically lower values (52.20 ± 6.37 t/ha). Similarly to SOC, C-SOC was also significantly affected by elevation with the highest values from 600 to 900 m asl (70.46 ± 4.23 t/ha).

**Table 2:** Soil organic carbon density (C-SOC), above-ground biomass carbon density (C-ABGB), below-ground biomass carbon density (C-BGB), living plant biomass carbon density (C-LPB), litter carbon density (C-Litter), total carbon density (C-TOT) in different soil types, grassland types and elevation. Contrasting letters denotes significant differences. P-values highlighted in bold are highly statistically significant (*P* < 0.001) and statistically significant (*P* < 0.05).

Soil type	C-SOC (t/ha)	C-ABGB (t/ha)	C-BGB (t/ha)	C-PLB (t/ha)	C-Litter (t/ha)	C-TOT (t/ha)
Fluvisols	95.52 ± 7.95 ^a^	1.16 ± 0.06 ^a^	3.63 ± 0.17 ^a^	4.80 ± 0.23 ^a^	0.34 ± 0.02 ^a^	100.64 ± 7.87 ^a^
Cambisols	52.22 ± 2.54 ^b^	0.85 ± 0.04 ^b^	2.68 ± 0.12 ^b^	3.54 ± 0.17 ^b^	0.25 ± 0.01^b^	55.43 ± 2.54 ^b^
Leptosols	52.20 ± 6.37 ^ab^	0.97 ± 0.08 ^ab^	3.03 ± 0.22 ^ab^	4.01 ± 0.29 ^ab^	0.29 ± 0.02 ^ab^	56.34 ± 6.20 ^ab^
Stagnosols	80.06 ± 8.40 ^a^	0.98 ± 0.11 ^ab^	3.10 ± 0.33 ^ab^	4.08 ± 0.44 ^ab^	0.29 ± 0.03 ^ab^	84.17 ± 7.90 ^a^
F value	17.42	6.35	6.87	6.75	5.88	19.22
P value	**< 0.01**	**0.006**	**< 0.001**	**< 0.001**	**0.001**	**< 0.001**
Grassland type						
Lowland hay meadows	68.68 ± 5.98 ^a^	0.97 ± 0.06 ^ab^	2.72 ± 0.16 ^bc^	3.69 ± 0.21^bc^	0.29 ± 0.02 ^b^	72.63 ± 6.00 ^a^
Mountain hay meadows	59.85 ± 3.49 ^a^	0.79 ± 0.05 ^b^	2.24 ± 0.16 ^c^	3.03 ± 0.22 ^c^	0.24 ± 0.02 ^ab^	62.69 ± 3.43 ^a^
Wet grasslands of sub-montane zones	71.04 ± 5.53 ^a^	1.07 ± 0.08 ^a^	4.31 ± 0.25 ^a^	5.38 ± 0.32 ^a^	0.32 ± 0.02 ^a^	77.20 ± 5.74 ^a^
Artificial grasslands	58.58 ± 12.90 ^a^	1.13 ± 0.07 ^a^	3.18 ± 0.22 ^b^	4.31 ± 0.30 ^ab^	0.34 ± 0.02 ^a^	63.14 ± 12.91 ^a^
F value	0.81	5.88	19.33	14.27	6.35	1.02
P value	0.493	**0.001**	**< 0.001**	**< 0.001**	**< 0.001**	0.389
Mountain range						
Great Fatra Mts.	56.85 ± 9.14 ^a^	1.22 ± 0.22 ^a^	3.40 ± 0.62 ^a^	4.62 ± 0.84 ^a^	0.36 ± 0.07 ^a^	63.83 ± 9.04 ^a^
Low Tatras Mts.	72.77 ± 5.62 ^a^	1.12 ± 0.07 ^a^	3.52 ± 0.31 ^a^	4.64 ± 0.38 ^a^	0.34 ± 0.02 ^a^	77.75 ± 5.65 ^a^
Slovak Paradise Mts.	89.69 ± 8.78 ^a^	0.41 ± 0.07 ^b^	1.14 ± 0.20 ^b^	1.54 ± 0.27 ^b^	0.12 ± 0.02 ^b^	91.35 ± 8.89 ^a^
Čergov Mts.	58.45 ± 4.17 ^a^	0.60 ± 0.05 ^b^	1.70 ± 0.14 ^b^	2.29 ± 0.19 ^b^	0.18 ± 0.02 ^b^	60.93 ± 4.20 ^a^
Slánské Hills Mts.	53.29 ± 5.19 ^a^	0.83 ± 0.08 ^c^	2.39 ± 0.25 ^c^	3.22 ± 0.33 ^c^	0.25 ± 0.03 ^c^	56.77 ± 5.25 ^a^
F value	2.52	34.39	29.96	32.76	34.39	2.69
P value	**0.046**	**< 0.001**	**< 0.001**	**< 0.001**	**< 0.001**	**0.036**
Elevation						
To 600 m asl	47.98 ± 4.88 ^b^	0.98 ± 0.18 ^a^	2.80 ± 0.51 ^a^	3.79 ± 0.51 ^a^	0.30 ± 0.05 ^a^	52.07 ± 4.86 ^b^
From 600 m asl to 900 m asl	70.46 ± 4.23 ^a^	0.90 ± 0.08 ^a^	2.75 ± 0.30 ^a^	3.65 ± 0.38 ^a^	0.27 ± 0.02 ^a^	74.38 ± 4.27 ^a^
Over 900 m asl	61.64 ± 7.04^a^	0.79 ± 0.12 ^a^	2.27 ± 0.36 ^a^	3.06 ± 0.48 ^a^	0.24 ± 0.04 ^a^	64.93 ± 7.11 ^a^
F value	4.14	1.49	1.18	1.22	1.49	4.06
P value	**0.018**	0.229	0.309	0.298	0.229	**0.020**
Interactions						
Soil Type x Grassland type						
F value	1.61	0.52	0.75	0.68	0.52	1.52
P value	0.123	0.856	0.662	0.729	0.856	0.152
Soil Type x Mountain range						
F value	3.29	8.84	8.21	8.89	8.84	2.89
P value	**0.002**	**< 0.001**	**< 0.001**	**< 0.001**	**< 0.001**	**0.006**
Soil Type x Elevation						
F value	2.21	0.69	0.25	0.32	0.67	2.23
P value	0.059	0.626	0.936	0.899	0.626	0.057

### Plant organic carbon density

The carbon density in living plant biomass varied noticeably across grassland types. On average, above-ground biomass contained 0.90 t/ha of carbon, with values ranging from 0.41 t/ha in the Čergov Mts. with Mountain hay meadows on the Cambisols to 1.95 t/ha in the Great Fatra Mts. where Lowland hay meadows occurred on the Leptosols.

Among the habitats, species rich Mountain hay meadows also showed the significantly (*P* < 0.05) lowest values in C-ABGB, C-BGB and C-PLB. This result is somewhat unexpected, as higher species richness is often associated with greater biomass, however, these meadows occurred at higher elevations with harsher climatic conditions, which likely constrained biomass accumulation. While above-ground carbon density was comparable in Wet grasslands and Artificial grasslands, Wet grasslands accumulated substantially more below-ground biomass carbon, resulting in the highest overall carbon density in living biomass among the studied habitats. The highest C-BGB was observed at Fluvisols (3.63 ± 0.17 t/ha). These values were 35% higher than C-BGB in Cambisols.

Similarly to the patterns observed in soil organic carbon, C-PLB was significantly affected by mountain range. Grassland types in the Low Tatra Mts. and the Veľká Fatra Mts. accumulated more than 4.6 t/ha carbon in living biomass, while the lowest carbon density in living biomass was in Lowland hay meadows observed of the Slovak Paradise Mts. (1.54 ± 0.43 t/ha). Moreover, the interaction between soil type and mountain range affected all components of living biomass carbon—C-ABGB, C-BGB, C-LPB, and C-Litter (Table 2).

### Total ecosystem carbon density

Total carbon density also varied significantly with soil type (F = 19.22, *p* < 0.001). The lowest values were found in Cambisols (55.43 ± 2.54 t/ha), followed Leptosol (56.34 ± 6.20 t/ha), while Stagnosol (84.17 ± 7.90 t/ha) and Fluvisol (100.64 ± 7.87 t/ha) stored markedly more carbon. In contrast, grassland habitat had no significant effect on the C-TOT (F = 1.02, *p* = 0.389). Across grassland habitats C-TOT ranged from 62.69 ± 3.43 in Mountain hay meadows to 77.20 ± 5.74 t/ha in Wet grasslands of sub-montane and montane zones. No significant interaction between soil and grassland types was detected (F = 1.52, *p* = 0.152).

### Dry matter yield and bioenergy potential

Patterns in biomass production and energy potential reflected similar ecological gradients. Mountain hay meadows produced the lowest DMY_ABG_ (1.47 ± 0.11 t/ha), while Lowland hay meadows, Wet grasslands, and Artificial grasslands exhibited comparable productivity. Much clearer differences emerged when evaluating bioenergy potential. All parameters - MEY, ASMY, and GEY followed the same order: Artificial grasslands > Wet grasslands > Lowland hay meadows > Mountain hay meadows. Artificial grasslands showed significantly higher of MEY (12.53 ± 0.80 GJ/ha), ASMY (338.63 ± 21.65 m^3^ CH_4_/ha) and GEY (41.60 ± 2.98 GJ/ha).

Soil type further shaped methane potential, with MEY being 54% higher in Fluvisols than in Cambisols (F = 10.83, *p* < 0.001). Regional patterns were also evident, grasslands in the Slovak Paradise Mts. yielded the lowest energy values, whereas those in the Great Fatra Mountains reached the highest. In these productive locations, Lowland hay meadows on Fluvisols and Leptosols yielded approximately twice as much as those on Cambisols in the Slovak Paradise Mts.

Although DMY_ABG_ and GEY tended to decrease with elevation, these trends were generally not statistically significant. MEY and ASMY showed a borderline decline with increasing altitude (F = 2.08, *p* = 0.063). Across all analyses, no significant interactions emerged among grassland type, soil type, and mountain range Table 3.

**Table 3:** Dry matter yield of above-ground biomass (DMY_AGB_), methane energy yield (MEY), area specific methane yield (ASMY), gross energy yield (GEY) in different soil types, grassland types and elevation. Contrasting letters denotes significant differences. P-values highlighted in bold are highly statistically significant (*P* < 0.001) and statistically significant (*P* < 0.05).

Soil type	DMY_ABG_ (t/ha)	MEY (GJ/ha)	ASMY (m^3^ CH_4_/ha)	GEY (GJ/ha)
Fluvisols	2.48 ± 0.12 ^a^	11.49 ± 0.63 ^a^	310.61 ± 17.13 ^a^	42.48 ± 2.21 ^a^
Cambisols	1.62 ± 0.08 ^b^	7.45 ± 0.41 ^b^	201.52 ± 11.31 ^b^	27.64 ± 1.46 ^b^
Leptosols	2.06 ± 0.17 ^ab^	9.74 ± 0.86 ^ab^	263.34 ± 23.28 ^ab^	35.31 ± 3.01 ^ab^
Stagnosols	2.00 ± 0.25 ^ab^	9.66 ± 1.26 ^ab^	261.08 ± 34.26 ^ab^	34.39 ± 4.43 ^ab^
F value	10.83	9.98	9.98	10.75
P value	**< 0.001**	**< 0.001**	**< 0.001**	**< 0.001**
Grassland type				
Lowland hay meadows	2.04 ± 0.10 ^a^	9.23 ± 0.46 ^b^	249.71 ± 12.66 ^b^	34.87 ± 1.74 ^a^
Mountain hay meadows	1.47 ± 0.10 ^b^	6.52 ± 0.48 ^c^	176.40 ± 13.19 ^c^	24.69 ± 1.81 ^b^
Wet grasslands of sub-montane zones	2.32 ± 0.18 ^a^	10.67 ± 0.83 ^ab^	288.61 ± 22.54 ^ab^	39.59 ± 3.10 ^a^
Artificial grasslands	2.36 ± 0.17 ^a^	12.53 ± 0.80 ^a^	338.63 ± 21.65 ^a^	41.60 ± 2.98 ^a^
F value	10.29	16.40	16.40	11.58
P value	**< 0.001**	**< 0.001**	**< 0.001**	**< 0.001**
Mountain range				
Great Fatra Mts.	2.58 ± 0.14 ^a^	12.18 ± 0.69 ^a^	329.22 ± 18.73 ^a^	44.39 ± 2.41 ^a^
Low Tatras Mts.	2.37 ± 0.07 ^a^	11.07 ± 0.37 ^a^	299.35 ± 10.08 ^a^	40.62 ± 1.30 ^a^
Slovak Paradise Mts.	0.86 ± 0.20 ^c^	3.90 ± 1.02 ^c^	105.53 ± 27.79 ^c^	14.73 ± 3.58 ^c^
Čergov Mts.	1.28 ± 0.08 ^c^	5.70 ± 0.44 ^c^	154.21 ± 11.96 ^c^	21.58 ± 1.54 ^c^
Slánské Hills Mts.	1.77 ± 0.11 ^b^	8.35 ± 0.55 ^b^	225.91 ± 15.07 ^b^	30.42 ± 1.94 ^b^
F value	34.40	33.26	32.26	34.65
P value	**< 0.001**	**< 0.001**	**< 0.001**	**< 0.001**
Elevation				
To 600 m asl	2.09 ± 0.15 ^a^	10.051 ± 0.77 ^a^	271.65 ± 20.957 ^a^	36.09 ± 2.75 ^a^
From 600 m asl to 900 m asl	1.91 ± 0.08 ^a^	8.82 ± 0.43 ^a^	238.40 ± 11.80 ^a^	32.68 ± 1.55 ^a^
Over 900 m asl	1.67 ± 0.18 ^a^	7.58 ± 0.89 ^a^	205.06 ± 24.20 ^a^	28.30 ± 3.17 ^a^
F value	1.49	2.19	2.19	1.72
P value	0.229	0.117	0.117	0.185
Interactions				
Soil Type x Grassland type				
F value	0.69	0.64	0.64	0.68
P value	0.713	0.751	0.751	0.729
Soil Type x Mountain range				
F value	3.84	3.76	3.76	3.81
P value	**< 0.001**	**< 0.001**	**< 0.001**	**< 0.001**
Soil Type x Elevation				
F value	0.223	0.25	0.25	0.23
P value	0.947	0.935	0.934	0.945
Grassland type x Mountain range				
F value	1.53	1.46	1.46	1.54
P value	0.176	0.201	0.200	0.174
Grassland type x Elevation				
F value	1.56	2.08	2.08	1.73
P value	0.169	0.063	0.063	0.123

### Inter-mountain comparison of carbon stock and bioenergy potential

According to the data of the Program for the Care of Protected Areas (https://www.minzp.sk/natura2000/chranene-vtacie-uzemia/programy-starostlivosti-chvu.html), grassland habitats across the five Western Carpathian regions covered a total of 33,560 ha (Table 4). Based on this area, the ecosystems stored an estimated 2.120 × 10^3^ tons of carbon, with an overwhelming 94% residing in the soil. The calculated biomethane production ranged from 6,606 GJ in the Slovak Paradise Mts. with Lowland hay meadows, to 122,888 GJ in the Low Tatra Mts., which had the largest area of grassland habitats. The theoretical energy potential exceeded 1,139 GJ. Overall, both carbon stock and energetic potential followed the same geographic gradient: Slovak Paradise Mts. < Čergov Mts. < Slánske Hills Mts. < Great Fatra Mts. < Low Tatra Mts.

**Table 4:** Soil organic carbon stock, total carbon stock, theoretical biomethane production and energy content of grassland area in Western Carpathians.

Mountain range	Grassland area (ha)	Soil organic carbon stock (t)	Total carbon stock (t)	Theoretical biomethane production (GJ)	Theoretical energy content (GJ)
Great Fatra Mts.	6,130	348,490	379,017	74,663	272,110
Low Tatras Mts.	11,101	807,819	863,102	122,888	450,922
Slovak Paradise Mts.	1,694	151,934	154,763	6,606	24,952
Čergov Mts.	6,148	359,350	374,597	35,043	132,673
Slánské Hills Mts.	8,493	452,591	482,147	70,916	258,357
Total	33,560	2,120,184	2,253,626	310,116	1,139,014

## Discussion

Mountain grasslands represent dynamic ecosystems shaped by a combination of harsh climatic conditions and diverse geological substrates^40^. These factors collectively influence soil development, nutrient availability, vegetation composition, and ultimately the capacity of these systems to store carbon and generate bioenergy.

The interactions between vegetation and soil nutrients provided further insight into mountain ecosystem functioning. Artificial grasslands showed higher plant-available P content what may be associated with a higher abundance of legumes (red clover and white clover) and their ability to improve P availability in the rhizosphere^41^. While phosphorus availability was uniformly low, plant-available Mg was exceptionally high and varied significantly with soil type and bedrock with the lowest values in Cambisols on volcanic and flysh parental rocks of the the Čergov Mts^42^. Moreover, a significant interaction between soil type and mountain range indicates that plant-available Mg patterns mirrored parent-material differences.

In general, the soils investigated in this study exhibited consistently low pH values, reflecting conditions typical of mountain environments. Mean soil pH ranged from 3.90 in carbonate-free Cambisols to 5.56 in Leptosols, closely aligning with values reported by Bohner for the Austrian Alps^43^. A polynomial relationship between elevation and pH has been reported elsewhere, with minimum acidity occurring at mid-elevations in several Cambisol taxa in Slovakia^44^. Given that soil pH strongly regulates nutrient availability, such patterns have important functional implications. The strong correspondence between plant-available Mg and pH emphasizes the influence of dolomitic and carbonate-rich substrates in shaping nutrient regimes. Such geological controls often dominate nutrient patterns in mountain ecosystems^45^.

Our study demonstrates that the mountain grasslands of the Western Carpathians constitute a significant carbon reservoir, with soil acting as the dominant storage pool. At the same time, these habitats show considerable spatial variability in carbon stocks, reflecting the complexity of mountain landscapes. Soil organic carbon is a key component of soil organic matter, and its levels naturally shift depending on soil type, intrinsic soil properties, climate, and a range of environmental influences. Mountain grasslands are generally rich in SOC, but the typically high spatial variability of mountain environments, combined with differences in management systems, makes their soil carbon content particularly heterogeneous^46^.

In our study, this variability became especially clear. Among the different mountain ranges and soil types, the Slovak Paradise and soils classified as Leptosols were particularly rich in topsoil SOC. This pattern aligns with findings from Poland^47^, where Rendzinas formed from calcium-carbonate-rich parent material were shown to accumulate more SOC than other soil types under the same vegetation cover.

Other authors^48,49^ have also noted that SOC tends to increase in the surface layers of mountain soils along climatic gradients, reinforcing the idea that cooler, high-elevation environments favour carbon accumulation. In contrast, the lowest SOC values in our dataset came from sites located below 600 m asl., where warmer temperatures likely contribute to faster organic matter decomposition and, consequently, reduced carbon storage^50^.

SOC density varied markedly with both soil type and elevation. In our study, fine-textured Fluvisols contained substantially more SOC than Leptosols, which are known for their highly skeletal character^51^. While one European study reported no meaningful differences in carbon storage between shallow Leptosols and deeply developed Cambisols^52^, research in the western Italian Alps^53^ found the opposite: Leptosols held the lowest SOC stocks when compared to Cambisols. Our results indicate that the largest SOC stocks occurred in Fluvisols, highlighting the key role of alluvial soils for long-term carbon storage. This is consistent with long-term observations from Switzerland^54^, where Fluvisols consistently maintained high organic matter content despite the dynamic nature of alluvial processes.

Several studies showed that both management practices and the type of grassland can strongly influence soil organic carbon density^55^. Leifeld et al.^56^ stated that soil organic carbon density of permanent grasslands under favourable conditions in Switzerland was 16% and 6% higher than that of in temporary grassland and permanent grassland in less favourable environments. Similarly, Abdalla et al.^57^ demonstrated that rotational grazing and fertilization in South Africa boosted SOC density by 60% and 50%, respectively, highlighting just how responsive soil carbon stocks can be to management interventions.

In our study, grassland habitat type did not significantly affect soil organic carbon stock, probably because cutting without any fertilization or nutrient application was the only management practice of the Carpathian grassland habitats studied. Our results therefore align with research from Western France on Cambisols^58^, where mown grasslands were found to store less SOC than grazed areas due to lower carbon inputs and reduced biomass diversity.

Across the sites we studied, the carbon density of living plant biomass showed considerable variation. Among soil types, on average, the carbon density of living plant biomass ranged from 3.54 t/ha for Cambisols to 4.80 t/ha for Fluvisols, while among habitats while among habitats. Several studies^59,60^ have demonstrated that below-ground biomass represents the major source of new soil carbon, whereas the role of above-ground biomass remains less clear. Tripolskaja et al.^61^ found that SOC in the 0–50 cm layer correlated more strongly with carbon accumulated in roots (*r* = 0.62) than with shoot biomass (*r* = 0.41), while Pan et al.^62^ further estimated that roots may contribute up to 60–80% of new carbon inputs in grassland ecosystems. In our study, however, the higher carbon density in the Wet grasslands of the sub-montane zone appeared to positively influence SOC levels. This suggests that under favorable conditions, above-ground litter inputs may also play a meaningful role in carbon accumulation. Overall, living plant biomass contributed only a small proportion of total ecosystem carbon at 15 cm soil depth: 4–6% in both Mountain hay meadows and Wet sub-montane grasslands, with below-ground biomass contributing an additional 3–5%. These findings are consistent with Liu et al.^63^, who reported in alpine meadows of the Qinghai Plateau that soil constitutes the largest carbon reservoir, with plant biomass accounting for merely 7% of the total carbon density. Similar conclusions were reached by Malhotra et al.^64^, who emphasized the importance of root-derived carbon inputs, demonstrating a strong positive relationship between fine-root carbon and SOC stocks across grassland ecosystems. Collectively, these studies highlight the critical role of below-ground processes in maintaining soil carbon pools, especially in environments where climate imposes strict limits on plant productivity.

In addition to being a carbon stock, grassland habitats can be used as a source of bioenergy. Our results show that this energy potential varies widely across grassland types, soil types, and mountain ranges. Among the grassland types studied, DMY_ABG_ did not show significant variations between Artificial grasslands, Wet grasslands of sub-montane zones and Lowland hay meadows. The lower DMY_ABG_ values observed in Mountain hay meadows align with findings from Mezule et al.^65^, who reported that average grassland productivity ranges from as little as 1.0 t/ha in xeric sand calcareous grasslands to as much as 6.0 t/ha in Lowland hay meadows.

While biomass yields were relatively similar across several habitat types, the differences became far more pronounced when evaluating biomethane production. Artificial grasslands produced significantly higher MEY, ASMY and GEY. This indicates that, the biochemical composition of the biomass plays a key role in determining bioenergy performance^66^. This pattern reflects conclusions by Prochnow et al.^24^, who highlighted that substrate quality, particularly fibre and lignin content, is a decisive factor influencing anaerobic digestion efficiency in semi-natural grasslands.

Soil type was found to be a significant factor influencing energy yield. Fluvisols supported 54% higher MEY compared to Cambisols, likely due to better nutrient availability. Our results are consistent with Segura et al.^67^, who reported that alluvial soils tend to promote more productive and digestible plant growth, making them especially suitable for anaerobic digestion. It should be emphasized that these values represent theoretical energy potential. In mountain regions, steep slopes, limited mechanization, and high transport costs substantially constrain the amount of biomass that can be practically harvested and used for energy production^68^.

Furthermore, biomass extraction for bioenergy can generate both positive and negative environmental impacts. Harvesting grasslands that were previously unmanaged may enhance soil quality, increase plant diversity, and provide habitat advantages for some invertebrates, while simultaneously supplying renewable energy. However, when management becomes more intensive, with frequent cutting or biomass removal, the multifunctionality of grasslands may decline. Such practices can negatively affect biodiversity and reduce the broader ecosystem services these landscapes provide^69^.

## Conclusions

Mountain grasslands provide a well-known set of essential ecosystem services. We focused on some of these important services, namely assessing carbon stock in soil, above-ground biomass and below-ground biomass. The relationship between soil chemical properties and carbon stock was also investigated. To prevent grassland degradation and biodiversity loss, it was established that biomass can also be used as biogas or biofuels, thus contributing to reducing dependence on fossil fuels and mitigating climate change.

We performed 98 samplings, and this allowed us to evaluate the effects of soil, grassland type and elevation on carbon stock and energy potential. The study sites confirmed that the soils of the Western Carpathians grasslands can be considered an important carbon reserve.

The main results showed that of the total carbon stock of mountain ecosystems, equal to 2.120 × 10^3^ tons, can be considered important and that as 94% of it is stored in soil. Soil type significantly affected the carbon density where Fluvisols showed the highest values (100.64 ± 7.87 t/ha). The soil organic carbon density was also significantly affected by elevation, with the highest values between 600 and 900 m asl (33.53 ± 3.28 t/ha). The calculated biomethane production varied very much from 6,606 GJ to 122,888 G. These large differences are due not only to the amount of biomass, but mainly to its physical-chemical composition.

We must also recognize some limitations of our research. In fact, although that it must be noted that larger part of the grassland plant roots is concentrated in the top 15 cm of soil and these data constitute a good starting point, having data on deeper soil levels can provide a more comprehensive understanding of carbon dynamics and sequestration in these ecosystems. This important extent of research should be continued.

From an energetic perspective, the potential biomethane production derived from grassland biomass provides an interesting insight into possible sustainable energy use. However, while theoretical estimates may suggest substantial energetic potential, practical implementation would require careful consideration of ecological sustainability, biodiversity protection, and technical feasibility.

Future research should focus on assessing the impact of elevation, climatic conditions and different land management practices on both carbon sequestration and energy outcomes. Finally, it should be noted that providing stakeholders and policy makers with information on soil characteristics, carbon sink and energy-providing capacities of mountain grasslands will be crucial for Slovak agricultural and environmental policy.

## Data Availability

The data that has been used is confidential. Data on soil type and soil texture were retrieved from a publicly available digital map, the Landscape Atlas of the Slovak Republic was used (https://app.sazp.sk/atlassr/).
